# Modelling the potential impact of climate change on the productivity of soybean in the Nigeria Savannas

**DOI:** 10.1371/journal.pone.0313786

**Published:** 2025-03-19

**Authors:** Jenneh F. Bebeley, Abdullahi I. Tofa, Alpha Y. Kamara, Jibrin M. Jibrin, Reuben Solomon, Musibau A. Adeleke, Lucky O. Omoigui, Osagie B. Eseigbe, Helen Peter-Jerome, Temitope D. Ademulegun

**Affiliations:** International Institute of Tropical Agriculture (IITA), Ibadan, Oyo State, Nigeria; Covenant University, NIGERIA

## Abstract

A well-calibrated and evaluated GROPGRO module of the Decision Support System for Agro-technological Transfer (DSSAT) was used to simulate productivity of soybean in northern Nigeria under climate change. Both historical (1990–2019) and projected climate scenarios from 5 general circulation models (GCMs) under two representative concentration pathways (RCP 4.5 and RCP 8.5) in the mid-century (2040–2069) and end of the century (2070–2099) periods were used. Depending on climate scenario, the minimum temperature is expected to rise by 1.7–4.4^o^C at Kano in the Sudan savanna (SS) agroecological zone (AEZ) and 1.4–4.0^o^C at Zaria in the northern Guinea savanna (NGS) AEZ, while maximum temperatures are projected to increase by 1.7–4.1^o^C in the SS and 1.3–3.6^o^C in the NGS. Seasonal average rainfall will increase by 4.8–14.5% in the SS and decrease by 2.6–3.8% in the NGS, relative to the baseline climate. The model predicted delaying trends for days to flowering and maturity for both varieties in all climate scenarios in the two AEZs. Despite the delay in flowering and increase in crop cycle length, climate change will result in grain yield reduction in most of the future scenarios. Across location, variety and time slice, the grain yield will decline by between 8.4 and 23.6% under RCP4.5 scenario, with much higher decline by between 28.7 and 51.4% under RCP 8.5 scenario. However, using the early maturing variety can reduce the adverse effects of climate change on grain yield. On average, the yield of the early-maturing TGX1835-10E is predicted to be 15.2% higher under RCP4.5 scenario and up to 21.7% under RCP8.5 than that of the medium-maturing TGX1951-3F for both centuries in the SS AEZ. In the NGS, the average yield of TGX1835-10E is predicted to be 9.0% and 7.5% higher than that of TGX1951-3F under RCP4.5 and RCP8.5 scenarios, respectively. Using early-maturing soybean varieties is a key management strategy to boost the resilience of soybean production in Nigeria’s savannas under climate change conditions.

## 1. Introduction

Soybean (*Glycine max* [L.] Merr.) production in the Nigeria savannas has been increasing rapidly due to its importance as a major cash crop for rural households and high demand for processing at industrial level [1]. Nigeria is the second largest producer of soybean in Africa after South Africa [2]. Soybean improves soil fertility in cereal-dominated cropping systems through biological nitrogen fixing [3,4]. It also helps to reduce parasitic weed infestations in crop fields when incorporated in crop rotation [5,6]. Due to the high demand for feed for animals, soybean processing in Nigeria has expanded and exceeded that of groundnut [7], making soybean one of the most important oil crop in Nigeria. In West Africa, including Nigeria, soybean production is mostly carried out by smallholder farmers with little inputs. mainly in the savanna belt under rain fed conditions; hence, very low yields are obtained [8–10].

Despite increase in soybean production in Nigeria, the average yield is <1 ton ha^−1^, which is below the potential yield of >3 ton ha^−1^ [11] in Africa. Drought and high temperatures during flowering and grain-filling stages negatively affect the yield of soybean in Nigeria [12]. Additional constraints include pests and diseases, low soil fertility, and inadequate agronomic techniques [8,9,13]. Delays in the onset of the rainy season are becoming more common in the Nigeria savannas [14]. Long dry spells at the beginning, middle, and end of the rainy season are becoming increasingly common, even in the wetter Guinea savanna [15]. Because of variations in rainfall patterns, rain-fed crop production is becoming unpredictable and farmers in the savannas suffer more risks during crop production [16].

Soybean, a short-day plant, is cultivated in subtropical and temperate regions where weather conditions significantly influence crop production. Rising temperatures negatively affects soybean yield in the Nigeria savannas. According to Onat et al. [17], the optimum temperature required for different genotypes of soybean development range between 25 and 30 °C. Maximum temperatures exceeding 35 °C foster heat stress, which has a negative impact on soybean flowering and pod set [17]. Climate change is predicted to cause a 2–2.5 °C rise in temperature across Nigeria by 2050 [18], with an increase of up to 3.2 °C under a high scenario within the same century [19]. In addition, it is expected that there will be regional temperature variations, with a larger increase (4.5 °C) forecast for the northeast by 2100 [20]. Such excessive temperatures and droughts will have a negative influence on Nigerian agriculture and food security, which rely heavily on rain-fed crop production systems [21,22].

In Nigeria, there are several reports on the current and projected impact of climate change either in the form of rising temperatures or drought [23–29]. For example, Peter and Odjugo [29] have reported a 1.1 °C rise in mean temperature from 1901 to 2005. The Guinea savanna of Nigeria is experiencing a gradual increase in mean annual air temperature [23]. According to BNRCC [25] the temperature will increase by as much as 1°C in 2030, 2.3°C in 2060 and 3.7°C in 2090 in northern Nigeria. Tofa et al. [30] predicted that temperatures in the Nigerian savannas would rise by 1.7–2.4 °C by 2040–2069 and by 2.2–3.0 °C by 2070–2100 under the RCP4.5 scenario. However, under RCP8.5 they reported an increase between 2.2–2.9 °C in 2040–2069, and up to 3.9–5.0 °C in 2070–2100. They also predicted that rainfall would increase in the Sudan while decreasing in the Guinea savanna.

Future climatic changes are predicted to have a significant impact on soybean productivity, depending on how much temperature and CO_2_ differ from baseline scenarios [31,32]. For example, heat stress may decrease pollination, reduce grain filling, and contribute to sterility during reproduction [33]. Predictions from mechanistic and empirical models showed that additional warming above optimal temperature thresholds during the soybean-growing period could severely reduce grain yield [34]. Understanding the possible effects of climate change on plant growth and development will assist in the development of adaptation methods to offset these effects.

Previous climate change impacts studies in Nigeria mostly focused on cereal crops, but less on legumes. Although some studies have examined the impacts of future climate indices on soybean production and productivity in Africa [10,35–38], there is limited research specifically assessing the effects of climate change on soybean growth and yield in the Nigerian savannas. There is need to study the impacts of current and future climate change on soybean production. Such information can be used to determine the crop adaptation options. The objective of this study was to assess the impacts of climate change on the phenology, growth and grain yield of soybean in two contrasting environment of Nigeria savannas using the CROPGRO-Soybean model.

## 2. Materials and methods

### 2.1. Study area

**Fig 1:**
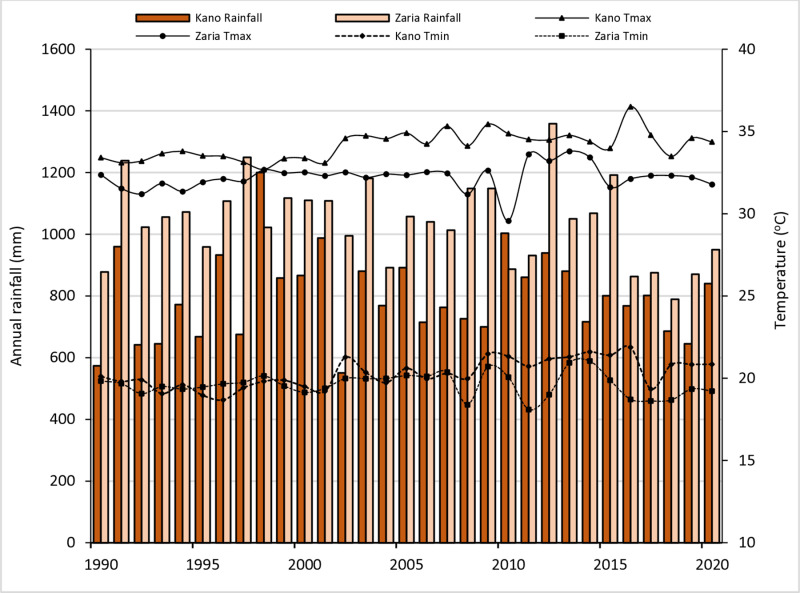
Thirty years’ historical (1990–2020) yearly average maximum and minimum temperatures and total annual rainfall at Kano in the Sudan savanna and Zaria in the northern Guinea savanna.

We conducted simulation studies in two locations in two different agroecological zones (AEZs) in Nigeria, Kano in the Sudan savanna (SS) and Zaria in the northern Guinea savanna (NGS) AEZs. Soybean is predominantly cultivated in the Guinea savanna of Nigeria, with Zaria, located in the NGS, representing a major production zone. However, due to the availability of early and medium-maturing varieties, soybean cultivation is expanding into the Sudan savanna, driven by the crop’s high demand in the industrial sector. The study area at Kano in the SS AEZ lies between longitude 8.516°E and latitude 11.516°N with elevation of 466 m above sea level. The SS has a single extended dry season followed by a rainy season that lasts from June to October. The baseline 30-year (1990–2020) mean annual rainfall was 824 mm, with average minimum and maximum temperatures of 20.3°C and 34.1°C in Kano. The soils are severely weathered and unstable, with little clay content [39]. According to the USDA soil taxonomy [40], the dominant soil class of the site which is Kano is Alfisol. The second location is Zaria in the NGS AEZ, situated within latitude 11.167°N and longitude 7.147°E and elevation of 702 m above sea level. The NGS experiences an extended dry season, spanning from November to April, and a single wet season occurring between May and October [41]. The mean annual rainfall from 1990 to 2020 was 1075 mm with an average minimum and maximum temperatures of 19.6°C and 32.2°C, respectively (Fig 1). In the NGS, the soils are categorised as leached ferruginous tropical soils with high clay concentration and drift debris [42]. According to USDA soil classification, this soil type is Typic Haplustalf [43]. In both the NGS and SS, agriculture is the primary occupation of the people. The dominant crops in the NGS include maize, sorghum, millet, groundnuts, and soybean, while in the SS, crops such as millet, sorghum, cowpea and groundnut are widely cultivated, with soybean gaining prominence due to increased demand [44].

### 2.2. Model Description and input data

The Decision Support Systems for Agrotechnology Transfer (DSSAT) [45] is a Cropping System Model that integrates a collection of computer programs and tools into a unified software package designed to streamline the utilization of crop simulation models in both research and decision-making processes [46]. Database management programs that control soil, weather, crop management, and experimental data support the software application. It is also equipped with utilities and application programs to simulate the growth, development, and yield as they relate to the dynamics between soil, plant, and atmosphere [47]. This study employed the CROPGRO-Soybean model within DSSAT to simulate the growth of two soybean varieties.

CROPGRO-Soybean is a widely used crop model designed to simulate the growth and development of soybean from planting to maturity, with a focus on physiological processes such as photosynthesis, respiration, transpiration, and root water intake. It operates on a daily time step, predicting yield by accounting for key factors like temperature, CO_2_, water supply, and management practices (e.g., planting date and row spacing) [48]. The model also integrates soil water dynamics, nutrient levels, and nitrogen balance, making it a comprehensive tool for evaluating soybean performance under various environmental conditions [48–50]. CROPGRO-Soybean requires minimal data inputs, including soil, weather, crop management, yield information, and cultivar coefficients [12,51]. It has been applied across different regions to simulate yield and assess soybean’s response to climate and management factors. For instance, Kamara et al. [52] used the model to estimate rainfed soybean production potential in northeast Nigeria, while Bebeley et al. [12] evaluated optimal sowing windows in the Nigerian savannas. Mekonnen et al. [50] quantified yield gaps in southwestern Ethiopia, and MacCarthy et al. [10] simulated soybean productivity under historical and projected climate scenarios.

In this study, the baseline observed daily weather data (maximum and minimum temperatures, solar radiation, and rainfall) from Kano and Zaria were used. The Nigeria Meteorological Agency (NIMET) provided the long-term (30-year) weather data from 1990 to 2020 for the two locations (Fig 1). The weather data for these two sites were entered into DSSAT Weatherman utility software to check for problems. Detailed soil profiling and characterization was done at each site. The soil profile generic horizons and types were classified according to FAO guidelines [53]. The physical and chemical properties of the soil, including soil organic carbon (total OC), total nitrogen (total N), soil pH in water, available phosphorus, and soil texture for each soil layer, were analysed and used for the study. Using the SBuild soil data tool in DSSAT, the model calculated the bulk density, drained upper limit (DUL), saturated upper limit (SAT), lower limit of plant-available soil water (LL), saturated hydraulic conductivity (Ksat), and root growth factor (RF). Bebeley et al. [12] reported the procedure and results for the physical and chemical features of soils used for calibration, evaluation and model applications.

### 2.3. Model calibration and evaluation

The DSSAT CROPGRO-Soybean model [47] used in the present study was calibrated and validated by Bebeley et al. [12]. The genetic coefficients for both varieties used in the study were adapted from Bebeley et al. [12] and are presented in Table 1. All calibration data to derive genetic coefficients were obtained from ten field experiments conducted across two sites at Bayero University Kano (BUK) and Dambatta between 2016 and 2019 in the Sudan savanna of Nigeria. The model was evaluated using independent datasets from field experiments conducted at Zaria and Doguwa during the 2016 cropping season and in 2017 and 2018 cropping seasons at Zaria only. Minimum crop datasets (flowering, maturity and grain yield) required for model calibration and evaluation were used. Bebeley et al. [12] also provided detailed information on the experiments and input data used for both calibration and evaluation. The findings of both model calibration and evaluation revealed good agreement in the model prediction of all tested parameters, as demonstrated by low root mean squared error (RMSE), a moderately high d-index, and strong model estimation efficiency [12]. This shows that the model has been successfully validated and may be applied as a decision-support tool for long-term scenario analysis in Nigeria’s savannas.

**Table 1:** Genetic coefficients for TGX1835-10E and TGX1951-3F soybean varieties used in the study (coefficients were adapted from [12]).

Coefficient	Definition	Unit	TGX1835-10E	TGX1951-3F
CSDL	Critical Short-day length below which reproductive development progresses with no day-length effect (for short-day plants)	hour	11.88	11.37
PPSEN	Slope of the relative response of development to photoperiod with time (positive for short-day plants)	1/hour	0.311	0.340
EM-FL	Time between plant emergence and flower appearance (R1)	pd^*^	29.94	27.84
FL-SH	Time between first flower and first pod (R3)	pd	7.000	6.000
FL-SD	Time between first flower and first seed (R5)	pd	20.85	14.35
SD-PM	Time between first seed (R5) and physiological maturity (R7)	pd	15.35	21.35
FL-LF	Time between first flower (R1) and of leaf expansion	pd	15.00	15.00
LFMAX	Maximum leaf photosynthesis rate at 30 °C, 350 vpm CO_2_, and high light	mg CO_2_/m^2^/s	1.016	1.016
SLAVR	Specific leaf area of cultivar under standard growth conditions	cm^2^/g	315.3	315.3
SIZLF	Maximum size of full leaf (three leaflets)	cm^2^	220.6	230.6
WTPSD	Maximum weight per seed	g	0.184	0.184
SFDUR	Seed filling duration for pod cohort at standard growth conditions	pd	20.27	18.45
SDPDV	Average seed per pod under standard growing conditions	#/pod	2.090	2.090
PODUR	Time required for cultivar to reach final pod load under optimal conditions	pd	10.00	10.00

* pd = photothermal days.

### 2.4 Future climate scenarios

The baseline climate for 30 years (1990–2020) was used to generate future climate scenarios based on the modified delta methodology, as described in the global Agricultural Model Intercomparison and Improvement Project (AgMIP) protocols [54]. Future climate scenarios for the mid-century (2040–2069) and end of the century (2070–2099) were generated under two representative concentration pathways (RCPs), namely, RCP 4.5 and RCP 8.5. The pathways represent two different greenhouse gas concentration trajectories used in climate modeling. We used the two RCPs in the study to capture a range of possible future climate conditions, from moderate to extreme scenarios. The delta-based technique was used to downscaled future climate scenarios. Future climatic scenarios under RCP 4.5 and RCP 8.5 predict increased CO_2_ concentrations of 499 and 571 ppm, respectively, compared to the current 380 ppm. The future daily rainfall, minimum, and maximum temperatures were derived by perturbing the daily baseline data using the delta factor method [55]. For this study, five contrasting GCMs from Fifth Coupled Model Inter-comparison Project (CMIP5) were used. These were Geophysical Fluid Dynamics Laboratory Earth System Model version 2M (GFDL-ESM2M), Hadley Centre Global Environment Model version 2 (HadGEM2-ES), Institute Pierre-Simon Laplace Climate Model for Phase 5 of CMIP with Low Resolution (IPSL-CM5A-LR), Model for Interdisciplinary Research On Climate (MICRO5) and Meteorological Research Institute Coupled Ocean–Atmosphere General Circulation Model 3 (MRI-CGCM3) were used in the analysis. These five GCMs based on their higher resolution (<2.5° longitude and latitude) and established ability to predict future climates in Africa and Nigeria [31,56–58]. Rainfall and temperature variations in the mid- and late-century relative to the baseline were assessed using GCM results.

### 2.5 Simulation protocols

The calibrated CROPGRO-Soybean model was used to access the impact of climate change on phenological parameters (flowering and maturity), grain yield and above ground biomass of the two soybean varieties in two agroecological zones (AEZs) of Nigeria. The effects of climate change on the phenology, above ground biomass and grain yield of each variety were simulated using 30-year baseline weather data from 1990 to 2020, with the baseline atmospheric CO_2_ of 380 ppm. Future climate scenarios, as described in Section 2.4, were utilized for the projected climate data. Two soybean varieties TGX1835-10E and TGX1951-3F that represent early and medium maturing varieties, respectively, were used. Long-term simulations were carried out in Kano on an Alfisol soil, representing the SS AEZ, and in Zaria on a Typic Haplustalf soil, representing the NGS AEZ. The planting date was set on June 20 in the SS and June 30 in the NGS, following recommended practices due to the variability in seasons, especially the establishment and cessation of rainfall in the study areas. The plant population was based on the national recommendation of 53.3 plants per square metre. A consistent inter-row spacing of 75 cm and planting depth of 4 cm were maintained in the model. For the simulations, the model was set to supply 17.3 kg P ha^−1^ at sowing, using triple superphosphate fertilizer material. The impact of climate scenarios on soybean phenological parameters (in days), grain yield and above ground biomass (in %) were compiled, and the relative change from the baseline was computed using Eq 1.


$$\Delta PYA=\frac{{}_{PYA_{future}-PYA_{baseline}}}{YPA_{baseline}}$$ (1)

*Where ΔPYA change in phenology, yield or above ground biomass due to climate change, PYA*_*future*_ *and PYA*_*baseline*_ *are values obtained under future climate scenarios and baseline weather conditions, respectively. All parameter changes mentioned under climate scenarios are ensemble values from five GCMs.*

## 3. Results

### 3.1. Projected climate changes in the study areas

The GCM ensemble for future climate projections in the study areas showed that both mean annual minimum and maximum temperatures would increase by mid and end-of-centuries under both RCP4.5 and RCP8.5 scenarios (Table 2). In Kano in the SS AEZ, the projection result showed that mean annual minimum temperature would increase by 1.7 °C under RCP4.5 and 2.7 °C under RCP8.5 by mid-century, as compared to the baseline period. Similarly, the mean annual maximum temperature may rise by 1.7 °C and 2.4 °C by mid-century under RCP4.5 and RCP8.5 scenarios, respectively. Projection for end-of-century period also showed that mean annual minimum temperature would increase by 2.3 °C and 4.4 °C under RCP4.5 and RCP8.5 scenarios, respectively. The mean annual maximum temperatures are projected to increase by 2.2 °C and 4.1 °C by end-of-century under RCP4.5 and RCP8.5 scenarios, respectively (Table 2).

**Table 2:** Changes in rainfall, maximum and minimum temperature for future periods relative to baseline mean of 1990–2020 under RCP 4.5 and RCP 8.5 scenarios in Kano and Zaria of Nigeria.

	Rainfall		Temperature	
			Maximum	Minimum
	(mm)	%	(°C)	(°C)
	Kano in SS			
Baseline mean	824	–	34.1	20.3
*Mid-Century*				
RCP4.5	64.6	7.3	1.7	1.7
RCP8.5	87.6	9.6	2.4	2.7
*End-of-Century*				
RCP4.5	41.8	4.8	2.2	2.3
RCP8.5	139.2	14.5	4.1	4.4
	Zaria in NGS			
Baseline mean	1075.0	–	32.2	19.6
*Mid-Century*				
RCP4.5	−31.8	−3.0	1.3	1.4
RCP8.5	−27.2	−2.6	1.9	2.3
*End-of-Century*				
RCP4.5	−39.4	−3.8	1.8	1.9
RCP8.5	−29.8	−2.9	3.6	4.0

In Zaria, located in the NGS AEZ, the projections indicate that the mean annual minimum temperature is likely to rise by 1.4 °C and 2.3 °C by mid-century under the RCP4.5 and RCP8.5 scenarios, respectively, compared to the baseline period. The mean annual maximum temperature is expected to increase by approximately 1.3 °C and 1.9 °C for RCP4.5 and RCP8.5 scenarios, respectively. By the end-of-century period, the mean annual minimum temperature is projected to increase by 1.9 °C and 4.0 °C for RCP4.5 and RCP8.5 scenarios, respectively. Similarly, maximum temperature is expected to rise by 1.8 °C and 3.6 °C under the RCP4.5 and RCP8.5 scenarios, respectively (Table 2).

The analysis of the projections indicates that in Kano in the SS, the mean annual total rainfall is projected to increase by approximately 65 mm (7.3%) and 88 mm (9.6%) by mid-century under the RCP4.5 and RCP8.5 scenarios, respectively. By the end of the century, it is anticipated to increase by 42 mm (4.8%) under RCP4.5 scenario and 139 mm (14.5%) under RCP8.5 scenario (Table 2). Conversely, in Zaria, the mean annual total rainfall is predicted to decrease by about 32 mm (3.0%) and 27 mm (2.6%) by mid-century under RCP4.5 and RCP8.5 scenarios, respectively. By the end of the century, it is expected to decrease by 39 mm (3.8%) and 30 mm (2.9%) under the respective scenarios (Table 2).

### 3.2 Impact of projected climate change on soybean phenology

**Fig 2:**
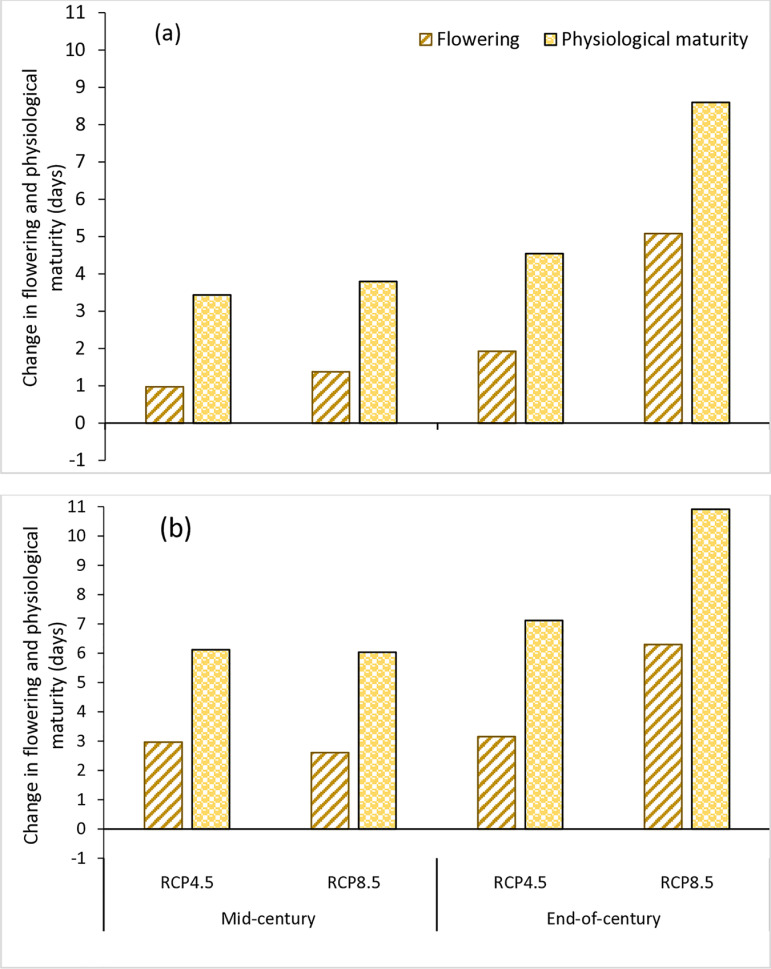
Change in flowering and physiological maturity (days) of TGX1835-10E (a) and TGX1951-3F (b) in mid-century (2040–2069) and end-of-century (2070–2099) under RCP4.5 and RCP8.5 scenarios in relative to the base period (1990–2019) at Kano, in the SS, Nigeria.

**Fig 3:**
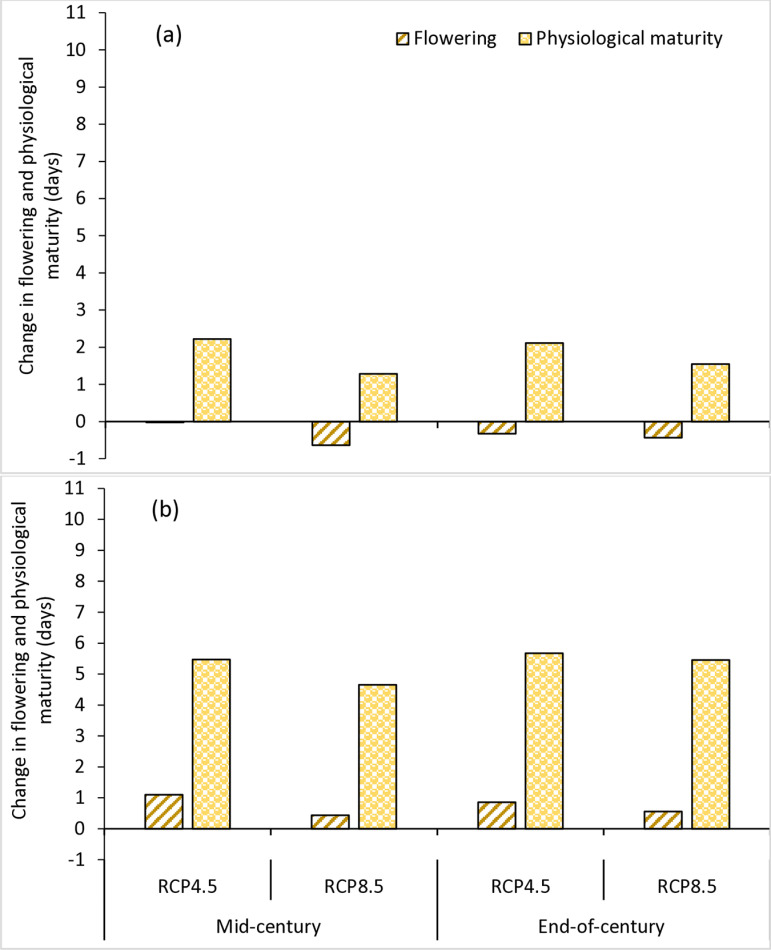
Change in flowering and physiological maturity (days) of TGX1835-10E (a) and TGX1951-3F (b) in mid-century (2040–2069) and end-of-century (2070–2099) under RCP4.5 and RCP8.5 scenarios in relative to the base period (1990–2019) at Zaria, in the NGS, Nigeria.

The projected climate change impact on the phenology of soybean varieties in the AEZs is presented in Figs 2 and 3. The results showed that the impact would vary with climate scenarios, AEZ and variety. At Kano in the SS, the number of days to 50% flowering would increase by 1 and 1.4 days for TGX1835-10E under RCP4.5 and RCP8.5 scenarios, respectively in the mid-century. For TGX1951-3F, the number of days to flowering would increase by 3.0 days under RCP4.5 and 2.6 days under RCP8.5 scenarios. At the end of the century, the number of days to flowering would increase by 1.9 and 5.1 days for TGX1835-10E under RCP4.5 and RCP8.5 scenarios, respectively. For TGX1951-3F, the number of days to flowering would increase by 3.2 days under RCP4.5 and 6.3 days under RCP8.5 scenarios (Fig 2). At Zaria in the NGS, the results indicate that the number of days to flowering will not be affected significantly for both varieties. In this AEZ, days to flowering would change by 0.03 and −0.6 days for TGX1835-10E under RCP4.5 and RCP8.5 scenarios, respectively. For TGX1951-3F, the number of days to flowering would increase by 1.1 days under RCP4.5 and 0.4 days under RCP8.5 scenarios. At the end of the century, the number days to flowering would reduce by −0.3 and −0.4 days for TGX1835-10E under RCP4.5 and RCP8.5 scenarios, respectively. For TGX1951-3F, the number of days to flowering would increase by 0.9 days under RCP4.5 and 0.6 days under RCP8.5 scenarios (Fig 3). 

**Fig 4:**
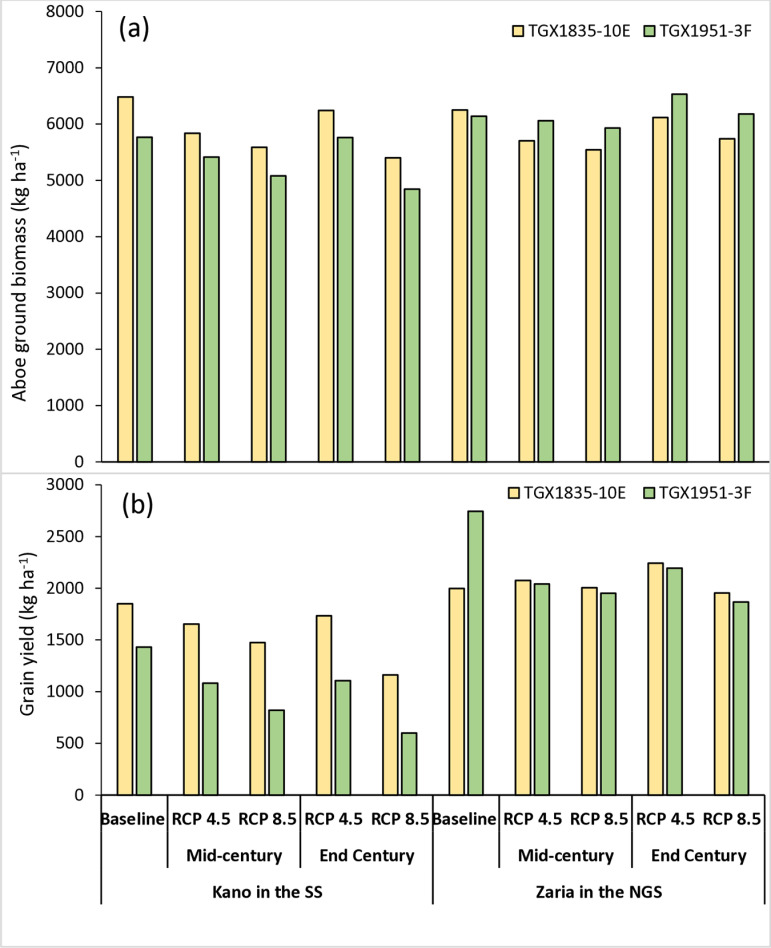
Average simulated dry matter (a) and grain yield (b) of soybean for baseline (1990–2020) and future scenarios in mid-century (2040–2069) and end-of-century (2070–2099) under RCP4.5 and RCP8.5 scenarios in the study sites.

The projected climate change impact on the physiological maturity of soybean varieties in the AEZs is presented in Figs 2 and 3. The results indicate that the number of days to physiological maturity will increase under all climate scenarios for both varieties in the two AEZs. In Kano, located in the SS AEZ, the number of days to maturity will increase in the mid-century by 3.4 and 3.8 days for TGX1835-10E under RCP4.5 and RCP8.5 scenarios, respectively. For TGX1951-3F, the number of days to physiological maturity would increase by 6.1 days under RCP4.5 and 6.0 days under RCP8.5 scenarios. By the end of the century, the number of days to maturity would increase by 4.5 and 8.6 days for TGX1835-10E under RCP4.5 and RCP8.5 scenarios, respectively. For TGX1951-3F, the number of days to maturity would increase by 7.1 days under RCP4.5 and 10.9 days under RCP8.5 scenarios (Fig 2). At Zaria, situated in the NGS AEZ, the results indicate that in the mid-century, the number of days to maturity would increase by 2.2 and 1.3 days for TGX1835-10E under RCP4.5 and RCP8.5 scenarios, respectively. For TGX1951-3F, the number of days to maturity would increase by 5.5 days under RCP4.5 and 4.7 days under RCP8.5 scenarios. At the end of the century, the number of days to maturity would increase by 2.1 and 1.5 days for TGX1835-10E under RCP4.5 and RCP8.5 scenarios, respectively. For TGX1951-3F, maturity would increase by 5.7 days under RCP4.5 and 5.5 days under RCP8.5 scenarios (Fig 3). 

### 3.3. Projected impact of climate change on above ground biomass and grain yield

**Fig 5:**
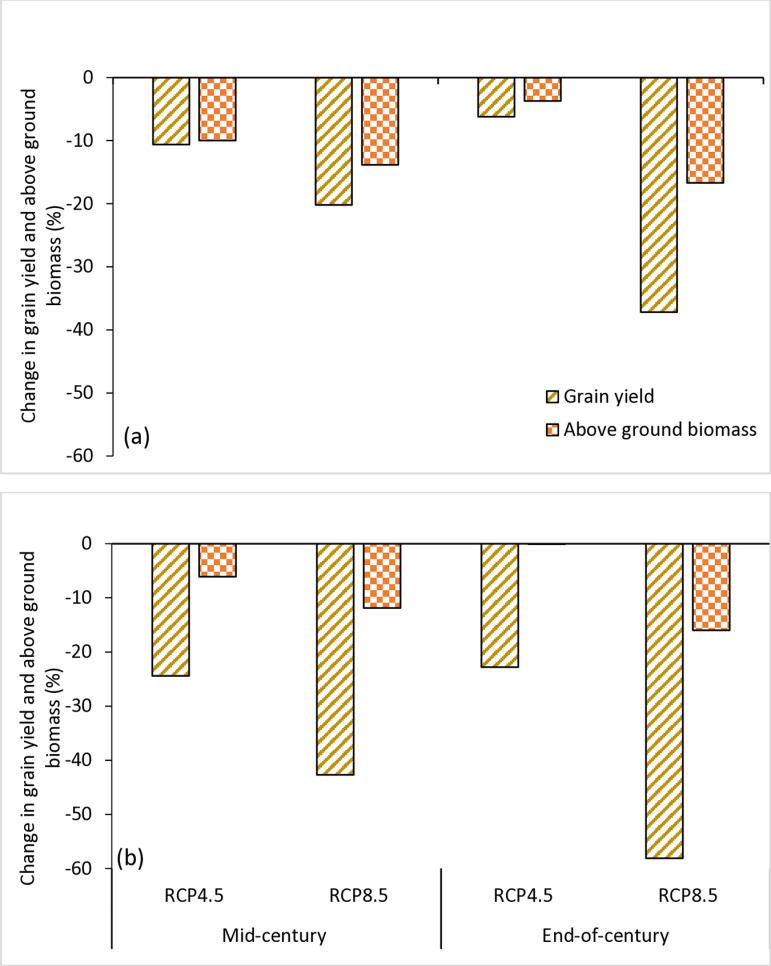
Change in grain yield and above ground biomass (days) of TGX1835-10E (a) and TGX1951-3F (b) in mid-century (2040–2069) and end-of-century (2070–2099) under RCP4.5 and RCP8.5 scenarios in relative to the base period (1990–2019) at Kano, in the SS, Nigeria.

**Fig 6:**
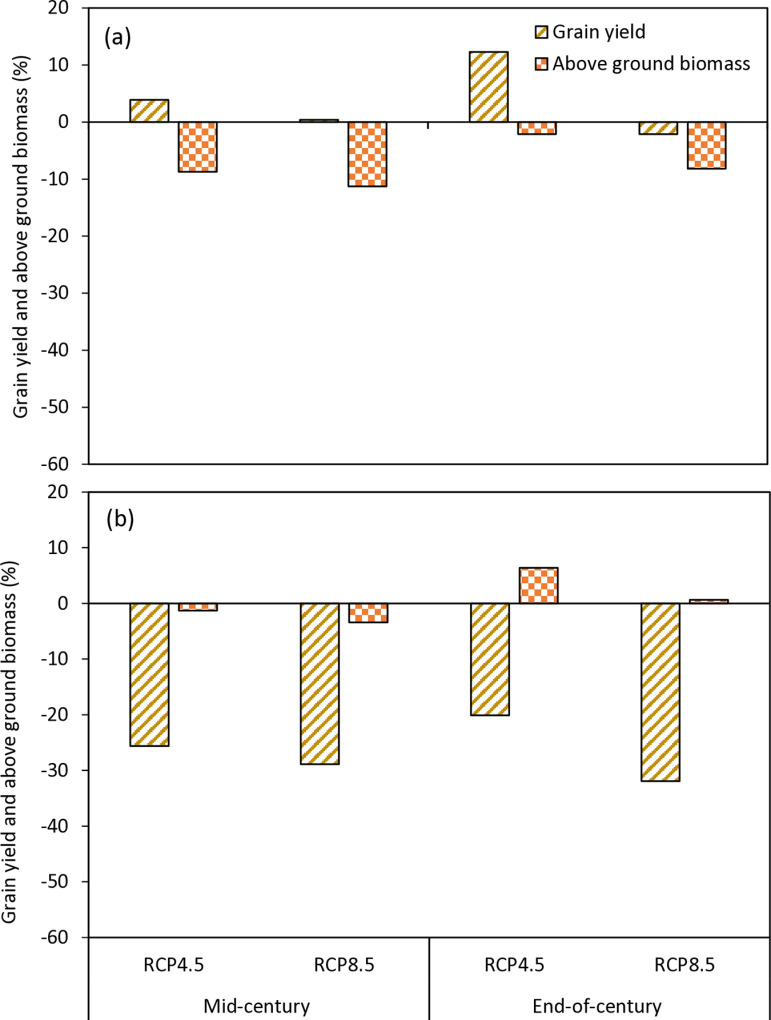
Change in grain yield and above ground biomass (days) of TGX1835-10E (a) and TGX1951-3F (b) in mid-century (2040–2069) and end-of-century (2070–2099) under RCP4.5 and RCP8.5 scenarios in relative to the base period (1990–2019) at Zaria, in the NGS, Nigeria.

Fig 4a shows the simulated above ground biomass, excluding roots, under the RCP4.5 and RCP8.5 scenarios in the two AEZs. In the SS, the baseline above ground biomass was 6481 kg ha^−1^ for TGX1835-10E and 5765 kg ha^−1^ for TGX1951-3F. The above ground biomass of TGX1835-10E would decrease to 5835 and 6240 kg ha^−1^, respectively, for the mid- and end-of-century periods under RCP4.5 scenario. This corresponds to a decrease of 10 and 3.7% respectively. Under the RCP8.5 scenario, the dry matter will decrease to 5585 and 5400 kg ha^−1^ for the respective periods corresponding to a decrease of 13.8 and 16.7%. The above ground biomass of TGX1951-3F will decrease to 5413 and 5760 kg ha^−1^, respectively, for mid- and end-of-century periods under the RCP4.5 scenario. This corresponds to a decrease of 6.1 and 0.1%, respectively. Under the RCP8.5 scenario, the dry matter recorded was 5080 kg ha^−1^ in the mid-century and 4844 kg ha^−1^ at the end of the century, which corresponds to decrease of 11.9 and 16.0%, respectively, compared to baseline (Fig 5). In the NGS, the simulated baseline above ground biomass is 6251 kg ha^−1^ for TGX1835-10E and 6139 kg ha^−1^ for TGX1951-3F. The predicted simulated above ground biomass of TGX1835-10E are 5705 and 6118 kg ha^−1^, respectively, for mid- and end-of-century periods under the RCP4.5 Scenario corresponding to reductions of 8.7 and 2.1%, respectively. Under RCP8.5, the projected dry matter was 5545 and 5740 kg ha^−1^ for the mid and end of century which corresponds to decreases of 11.3 and 8.2%, respectively. The projected above ground biomass of TGX1951-3F are 6060 and 6532 kg ha^−1^, respectively, for mid- and end-of-century periods under the RCP4.5 scenario corresponding to a reduction of 1.3% and increase of 6.4%, respectively, relative to baseline above ground biomass. Under the RCP8.5, the dry matter was projected to be 5929 kg ha^−1^ in the mid-century and 6178 kg ha^−1^ at the end-of-century period (Fig 5). corresponding to above ground biomass decrease of 3.4% and an increase of 0.6%, respectively, relative to baseline (Fig 6). 

Fig 4b shows simulated grain yield of soybean under the RCP4.5 and RCP8.5 scenarios in the two AEZs. In the Sudan savanna, the baseline grain yield is 1850 kg ha^−1^ for TGX1835-10E and 1432 kg ha^−1^ for TGX1951-3F. Under the RCP4.5 scenario, the simulated grain yields of TGX1835-10E are projected to be 1654 and 1734 kg ha^−1^ for the mid- and end-of-century periods, respectively, which correspond to 10.6 and 6.2%, decrease in yield, respectively. Under the RCP8.5 scenario, the grain yields are projected to be 1475 and 1162 kg ha^−1^ corresponding to a reduction in yield by 20.2 and 37.2%, for the mid- and end-of-century, respectively. The grain yields of TGX1951-3F are projected to be 1085 and 1106 kg ha^−1^ for mid- and end-of-century periods under RCP4.5 scenario. These correspond to 24.4 and 22.8% reduction in grain yield. Under RCP8.5 scenario, the projected grain yields are 820 kg ha^−1^ for mid-century and 600 kg ha^−1^ for end-of-century periods, corresponding to decreases in yield of 42.7 and 58.1%, respectively (Fig 5). 

In the northern Guinea savanna, the baseline grain yield are1997 kg ha^−1^ for TGX1835-10E and 2745 kg ha^−1^ for TGX1951-3F. Under RCP4.5 scenario, TGX1835-10E is projected to yield 2075 and 2242 kg ha^−1^ for mid- and end-of-century periods, respectively corresponding to 3.9 and 12.3% increase in yields, respectively. Under RCP8.5, the variety TGX1835-10E is expected to produce grain yield of 2004 kg ha^−1^ resulting into yield increase by 0.4% in the mid-century. The grain yield will decline to 1954 kg ha^−1^ at the end of the century corresponding to yield decrease of 2.1%. The grain yields of TGX1951-3F are projected to be 2042 and 2193 kg ha^−1^ for mid- and end-of-century periods under RCP4.5 corresponding to 25.6 and 20.1% yield decreases, respectively. Under RCP8.5, grain yield will decline to1952 and 1868 kg ha^−1^ for mid- and end-of-century periods, respectively, with corresponding reduction in yield of 28.9 and 31.9%, respectively (Fig 6). 

## 4. Discussion

### 4.1. Projected change in temperature and rainfall

Changes in temperature and rainfall have been projected to vary with location and AEZ consistent with the findings of Tofa et al. [14]. In both locations, the minimum and maximum temperatures would increase significantly in both the mid-century and the end of the century under RCP4.5 and RCP8.5 scenarios. Our results showed that the temperature changes vary spatially and increase from the NGS to the SS. However, the highest increase in temperature were projected under the RCP8.5 scenario compared to RCP4.5 scenario in both AEZs, which is in agreement with other findings in West Africa [31,59,60]. The results indicate that temperature will increase on average between 1.60°C in the NGS and 1.98°C in the SS under RCP4.5 and between 2.95 and 3.40°C in the respective AEZs under RCP8.5. This is consistent with Tofa et al. [30] who showed that temperature will increase on average between 2.3°C in the NGS and 2.6°C in the SS under RCP4.5 scenario corresponding to 3.6°C and 3.8°C under RCP8.5 scenario. Similarly, Jalloh et al. [18] predicted an increase of between 2 and 2.5 °C across the country by 2050, with a rise of up to 4 °C in the Sudan savanna [61]. The Intergovernmental Panel on Climate Change (IPCC) confirmed that global surface temperature would likely rise from 1.1°C to 6.4°C during the 21^st^ century [62]. In both AEZs, the night temperature (minimum temperature) shows a more pronounced increase compared to maximum temperatures under both RCPs. This finding corroborates the results of Dike et al. [63] and Tofa et al. [30] who showed that minimum temperature increases will be higher than those of maximum temperatures. The rise in minimum temperature will lead to an increase in daily mean temperatures and trigger the depletion of stored sugars in plants, which in turn will have adverse effects on the final crop yield [64,65]. The general increase in temperature emerges as a significant negative consequence of climate change, outweighing changes in precipitation patterns as indicated by climate models [66].

Our research revealed expected average rainfall increases of 6.1% and 12.1% under RCPs 4.5 and 8.5, respectively, in Kano in the SS AEZ, with an average decrease of –3.4% and –2.75% under RCPs 4.5 and 8.5 scenarios, respectively, in Zaria in the NGS AEZ. The increase in seasonal rainfall in SS and reduction in the NGS in this study is consistent with those predicted by Shiru et al. [67] and Tofa et al. [30]. Shiru et al. [67] projected rainfall increases of 5.5 to 6.9% under RCPs 4.5 and 8.5 scenarios, respectively, in Nigeria. Tofa et al. [30] predicted increase in rainfall by 6.9–20.1% in the Sudan savanna and by 1.9–6.6% in the Guinea savannas of Nigeria. In the savannas of northern Ghana, the percentage change in rainfall in Northern Ghana is expected to vary from –3.97% to 2.17% under RCP 4.5 and –7.21% to 9.51% under RCP 8.5 scenario [10]. The predicted increase in rainfall in the SS region could add to the risk of annual flooding, affecting agricultural performance. While the projected decrease in NGS may induce an early cessation of rain despite high cumulative rainfall or late-season drought, which could have a significant impact on soybean yield.

### 4.2. Impact of climate change on soybean phenology

The results of the simulation show that climate change will affect the phenology of soybean in the two AEZs and that the impact will vary depending on the climate scenario, variety, and AEZ. The model predicted delaying trends for both days to flowering and days to maturity for both varieties in all climate scenarios in the two AEZs, with much delay in the SS AEZ. The delay in phenology may be due to the very high temperatures predicted for these zones for both time slices. Soybean is a short-day crop, but its response to day length varies with variety and temperature. The crop exhibits high sensitivity to changes in both day length and ambient temperature with some studies suggesting that higher temperatures promote flowering [68–70]. However, contrasting findings indicate that elevated temperatures can delay flowering in soybeans [71,72]. The average baseline maximum temperatures of 34.1°C in the SS and 32.2°C in the NGS (Table 2) already exceed the optimal threshold of 30.0°C for soybean growth in Nigeria [73]. Tang et al. [74] observed that increasing temperatures from 25 to 30°C resulted in earlier flowering in two soybean varieties. However, further increase to 35°C notably postponed flowering compared to conditions at 25°C. According to Yang et al. [75], high night temperature above 29°C and heat can delay soybean maturity, as the interaction between photoperiod and temperature regulates flowering in soybeans. The highest delay in flowering and physiological maturity was under RCP8.5 scenario at the end of the century, aligning with the substantial temperature increases associated to high carbon emissions. Higher temperatures, leading to heat stress, significantly affect various aspects of soybean phenology, such as flowering time and the durations of vegetative and reproductive growth stages [76].

We also found that the changes in phenological parameters due to climate change were not similar for the two varieties in the two AEZs. In the SS AEZ, the delay in flowering could range from 1.0–5.1 days for TGX1835-10E and between 2.6–6.3 days for TGX1951-3F. While physiological maturity will delay between 3.4–8.6 days for TGX1835-10E and 6.0–10.9 days for TGX1951-3F. In the NGS AEZ, the results indicate that the number of days to flowering will not be affected significantly for both varieties and could change between ± 1 days because of lower increases in temperature. While physiological maturity is expected to delay between 1.3–2.2 days for TGX1835-10E and 4.7 and 5.7 days for TGX1951-3F. This could be associated with the different crop-cycle duration and the temperature variation observed between the two AEZs. Gong et al. [77] also reported that the phenology of soybean significantly varies with climatic conditions, and temperature is the most important among these climatic factors influencing the phenological parameters. According to Sadeghi and Niyaki [78], environmental conditions affect different soybean varieties with early maturing varieties being more sensitive to high temperatures compared to late-maturing varieties. For this reason, substantial variations among the soybean varieties were observed for both flowering and maturity.

### 4.3. Impact of climate change on soybean dry matter and grain yields

Results of our simulation confirm that climate change will constitute a major threat to soybean production in the Nigeria savannas. Despite the delay in flowering and increase in crop cycle length, the crop response to climate change will result in dry matter and grain yield decrease in most of the future scenarios. The decline may be due to reduction in yield components such as number of pods per plant, number of seeds and individual seed weights. Onat et al. [17] indicated that high temperatures reduced yield of soybean due to negative effects on yield components. According to Ogunkanmi et al. [36], increased atmospheric demand for water in a high temperature environment resulted in high evapotranspiration, leading to high transpiration, which most likely reduced photosynthetic activity of the plants, contributing to biomass and grain yield loss in soybean. Biomass and yield were drastically reduced due the combined effect of high temperature and drought.

Simulated dry matter and grain yields are projected to decrease in most future climate scenarios compared to the baseline in the AEZs. This is consistent with the findings of MacCarthy et al. [10] in the savannas of Ghana. From the results, the decline will be higher under RCP8.5 scenario than that under RCP 4.5 for both parameters. Irrespective of location, variety and time slice, the dry matter will decline between 2.2% and 6.9% under RCP4.5 with higher decline between 3.5% and 14.9% under RCP 8.5 scenario. Similarly, the grain yield will decline between 8.4% and 23.6% under RCP4.5, with much higher decline between 28.7% and 51.4% under RCP 8.5. Tofa et al. [30] projected that climate change will reduce grain yield of maize in the Nigeria savannas. They attributed the decline to increases in temperature and low rainfall in the SS and high temperatures in the NGS. MacCarthy et al. [10] projected 3 to 14% decline in grain yield of soybean across GCMs and RCPs in the savannas of northern Ghana due to climate change. Jumrani and Bhatia [79] reported that soybean seed yields decreased significantly when planted at high temperatures of 38–42 °C, with yield decreases of 42 and 64%, respectively. According to Amaka et al. [73], soybean dry matter and grain production improved when temperatures reached the ideal threshold value of 30 °C during the growing season. However, increasing temperatures by ≥4 °C lowered dry matter by 23.8% and grain production by 1% in Nigeria’s humid tropical climate.

The low grain yield of TGX1951-3F in the NGS despite the increases in dry matter may be due to low translocation of photosynthates from the leaves to the grain under high temperatures and project decrease in rainfall. The yield reductions predicted under the current study may be attributed to the increases in temperatures projected in the two AEZs. Consistent with our results, Liang et al. [80] simulated soybean yields in Harbin in northeast China using various climate change scenarios, and their results predicted a decrease of 10.6% on average across all RCP scenarios, with the highest decline under RCP 8.5. Tacarindua et al. [71] reported that above ground biomass, seed yield, and HI of soybean were consistently reduced by increasing temperature. The projected yield increase of TGX1835-10E by 3.9% and 12.3% under RCP 4.5 by mid-century and end of century, respectively, in the NGS, is likely attributed to early maturing trait, enabling it to complete its life cycle within a short period before the heat increases. Similarly, rainfall is anticipated to decrease under all climate scenarios in the NGS, which could pose challenges for the medium maturing variety. Compared to the baseline, it is expected that the dry matter will increase by 6.4% under RCP 4.5 for a medium maturing variety in the NGS. This increase could be attributed to a moderate rise in carbon dioxide levels to 499 ppm under RCP4.5 with moderate increase in temperature when compared with that under RCP8.5, which is likely to stimulate greater photosynthate production, leading to increased biomass accumulation in the crop. Drag et al. [81] observed that initially, soybean biomass accumulation would increase with rising carbon dioxide levels, but this effect diminishes as carbon dioxide levels exceed 900 ppm. Lenka et al. [82] studied the individual and combined effects of high CO_2_ and temperature on soybean growth and yield components in India. Their findings revealed a significant positive influence of elevated carbon dioxide and temperature on above ground biomass with an increase of 47% under elevation of both temperature and CO_2_ compared to ambient chamber.

According to our research, SS AEZ would experience a greater percentage reduction in dry matter and grain yield under all climate scenarios. According to our climate analysis, the SS AEZ is projected to experience higher temperatures than that of NGS AEZ. This will result to lower net photosynthetic carbon assimilation and biomass production as reported by Ruiz-Vera et al. [83]. During the soybean-growing season, high temperatures coupled with heat stress, particularly after flowering, would reduce the number of pods and seed weight, and consequently the grain production [79]. For soybean, the number of pods, the number of seeds per pod, and the seed weight are the primary component influencing grain yield [13,84]. Another impact of temperature is the increase in the night temperatures across the AEZ, which usually limits the normal crop performance due to high transpiration rate [74]. In comparison to the daytime temperature, the night temperature will rise by 0.2 °C in the SS AEZ and by 0.3 °C in the NGS AEZ across RCPs and periods. Previous studies have reported that high night temperature had a negative impact on soybean yield [71,75,85,86]. Tacarindua et al. [71] revealed that high night temperatures increased respiration-driven carbon losses and affected the flow of photosynthetic assimilation products from source organs to reservoirs, resulting in insufficient grain filling and, ultimately, a decrease in yield.

Although most future climate change scenarios indicate decreases in dry matter and grain yield, the response of two varieties varied under moderate and extreme representative concentration pathways (RCPs). Our research demonstrated that the projected reduction in dry matter would be less pronounced when utilizing the medium maturing variety (TGX1951-3F) compared to the early maturing variety (TGX1835-10E). However, the difference in dry matter between TGX1835-10E and TGX1951-3F was not statistically significant, ranging between 1–3% in the SS and 3-6% in the NGS among the climate scenarios. Nevertheless, adopting the early maturing variety (TGX1835-10E) can mitigate the adverse effects of climate change on grain yield. On average, the yield of the early-maturing variety TGX1835-10E was higher than that of the medium-maturing variety TGX1951-3F under all climate scenarios and time slices in both AEZs. The shorter growth period of TGX1835-10E could allow it to avoid exposure to excessively high temperatures during the critical reproductive phase. As a result, selecting the appropriate variety becomes essential in anticipation of future climatic conditions. Lawal et al. [87] documented that early maturing varieties have the potential to mitigate food and nutritional insecurity caused by terminal drought induced by climate change. However, the high yield reduction of 20.2% in the mid-century and 37.2% under RCP8.5 observed for the early maturing (TGX1835-10E) variety under RCP4.5 in the SS AEZ highlighted the need to breed soybean varieties that combine tolerance to drought and heat stress induced by elevated temperatures. While our findings provide valuable insights into the performance of soybean under the climate conditions of the selected soils, we recognize that soil variability across the entire savanna region may influence crop performance. Therefore, the results should be interpreted with an understanding of the specific soil contexts of the study, and further research may be needed to evaluate the soybean performance at scale using a broader soil conditions in the savanna zones in Nigeria.

## 5. Conclusions

In this study, we used the CROPGRO-Soybean model to evaluate the effects of climate change on soybean phenology, above ground biomass, and grain yield in Nigerian savannas. Climate change will influence the growth and yield of soybean in both Sudan and Guinea savanna of Nigeria. The model projected delayed trends in days to flowering and maturity for both soybean varieties across all climate scenarios in the two agro-ecological zones (AEZs). Climate change will result in dry matter and grain yield reduction in most of the future scenarios. On average, the grain yield will decline by between 8.4 and 23.6% under RCP4.5 scenario, with much higher decline by between 28.7 and 51.4% under RCP 8.5 scenario. The magnitude of this yield decrease may be due to the high temperature increase in the study area. Our results show that using the early-maturing TGX1835-10E variety may serve as a strategic management option for improving soybean productivity resilience under climate change in Nigeria savannas. The simulations suggest that, compared to the medium maturing, TGX1835-10E could yield an average of 15.2% higher under the RCP4.5 scenario and up to 21.7% under RCP8.5 across both centuries in the SS AEZ. Similarly, in the NGS, the early maturing variety is predicted to outperform TGX1951-3F by 9.0% and 7.5% under RCP4.5 and RCP8.5 scenarios, respectively, thereby reducing potential yield decrease under climate change. Therefore, the Government, Non-Governmental Organisations, and other development partners should help farmers to promote the use of early maturing soybean varieties in order to reduce the adverse effect of climate change on yield of soybean in the Nigeria savannas.
